# Rapid Isolation of Antibody from a Synthetic Human Antibody Library by Repeated Fluorescence-Activated Cell Sorting (FACS)

**DOI:** 10.1371/journal.pone.0108225

**Published:** 2014-10-10

**Authors:** Sung Sun Yim, Hyun Bae Bang, Young Hwan Kim, Yong Jae Lee, Gu Min Jeong, Ki Jun Jeong

**Affiliations:** 1 Department of Chemical and Biomolecular Engineering, Korea Advanced Institute of Science and Technology (KAIST) (BK21 plus program), Daejeon, Republic of Korea; 2 Institute for the BioCentury, KAIST, Daejeon, Republic of Korea; Naval Research Laboratory, United States of America

## Abstract

Antibodies and their derivatives are the most important agents in therapeutics and diagnostics. Even after the significant progress in the technology for antibody screening from huge libraries, it takes a long time to isolate an antibody, which prevents a prompt action against the spread of a disease. Here, we report a new strategy for isolating desired antibodies from a combinatorial library in one day by repeated fluorescence-activated cell sorting (FACS). First, we constructed a library of synthetic human antibody in which single-chain variable fragment (scFv) was expressed in the periplasm of *Escherichia coli*. After labeling the cells with fluorescent antigen probes, the highly fluorescent cells were sorted by using a high-speed cell sorter, and these cells were reused without regeneration in the next round of sorting. After repeating this sorting, the positive clones were completely enriched in several hours. Thus, we screened the library against three viral antigens, including the H1N1 influenza virus, Hepatitis B virus, and Foot-and-mouth disease virus. Finally, the potential antibody candidates, which show K_D_ values between 10 and 100 nM against the target antigens, could be successfully isolated even though the library was relatively small (∼10^6^). These results show that repeated FACS screening without regeneration of the sorted cells can be a powerful method when a rapid response to a spreading disease is required.

## Introduction

For the last two decades, monoclonal antibodies and antibody fragments have been proven to be effective as therapeutic and diagnostic agents, and have long been invaluable tools in various fields of biological research [1], [2]. For the development of antigen-specific antibodies, hybridoma technology that relies on animal immunization has been traditionally employed [3]. The recent progress in combinatorial technologies because of *in vitro* antibody repertoires and high-throughput screening methodologies has allowed the development of target-specific antibodies without animal immunization [4], [5]. In these technologies, various protein display systems including phage display, ribosome display, and cell-surface display, have been widely used for the initial isolation of antibodies specific to antigens from huge libraries, as well as for engineering the antibodies towards desired functions, e.g., enhanced affinity and higher thermostability. [6], [7], [8]. However, the most recent tools require repeated screenings in order to isolate potential candidates from the library, and consequently, they require relatively long time periods (several days to weeks) to complete the screening. The recent emergence and rapid dissemination of new viruses that cause serious human and animal diseases, such as SARS coronavirus, swine flu H1N1 virus, and avian influenza H5N1 virus, has raised world concerns. The development of new tools to quickly isolate antibodies against rapidly spreading infectious viruses for treatment as well as early diagnosis is urgently required.

Currently, fluorescence-activated cell sorting (FACS) has been used in high-throughput screening of huge libraries (generally bigger than 10^6^ cells) that are constructed in various display systems in bacteria or yeast as the host [6], [8]–[10]. The following strategy is usually used for screening a recombinant antibody library: (i) cultivation of library cells; (ii) fluorescent-antigen-peptide or protein labeling of the library cells; (iii) FACS sorting of the highly fluorescent population; (iv) regeneration of the sorted cells by regrowth or re-cloning of the sorted target genes; (v) repetition of steps i–iv until a highly fluorescent population is separated from the negative control population; and (vi) analysis of the individual clones. Among these steps, the step determining the screening time is the regeneration of the sorted cells (step iv). In all of the current screening strategies, the sorted cells need to be regenerated for the next round of sorting, which can be done by cultivating the cells for at least one day [6], [9], [10] or by re-cloning the genes, which takes several days [11]. In addition to the regeneration time, contamination of the sorted cells by non-specific clones also needs to be considered. During the cultivation for regeneration of sorted cells, differential growth rates among various clones (particularly non-specific clones) due to unregulated protein expression and differing cell viability can decrease the library screening efficiency, resulting in more rounds of sorting (longer duration) to isolate the potential antibody candidate [12].

**Figure 1:**
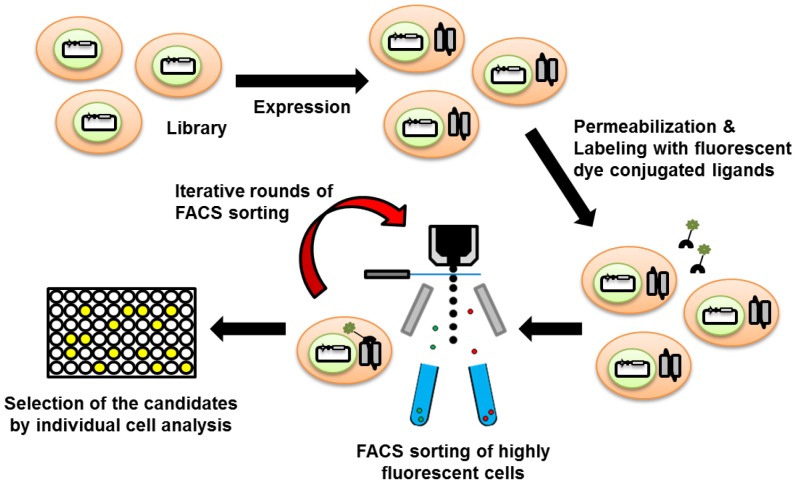
A schematic diagram showing the principle of repeated sorting strategy for isolation of high-affinity antibody. Positive population can be sorted from a large amount of previous library by FACS sorting, and the sorted sample become a sample for the next round of FACS sorting directly.

Herein, we report the development of a new high-throughput screening strategy based on *Escherichia coli* protein display and FACS sorting, which allows the simple and rapid isolation of potential candidates from a huge library in one day. First, we constructed the fully synthetic human antibody library in which antibody fragments (single-chain variable fragment, scFv) were produced in the periplasm of *E. coli*. After library cultivation and permeabilization, the cells were labeled with fluorescent antigen probes, and the highly fluorescent cells were sorted by using a high-speed cell sorter. Immediately after the first-round sorting, the sorted cells were reused in the next round of sorting, without regeneration of the sorted cells. This resorting was repeated until a highly fluorescent population became enriched as the major population and, using the high-speed FACS sorter, the best candidates could be isolated in one day. The overall strategy of this rapid screening is illustrated in the Figure 1. The proof of this concept was successfully demonstrated by the isolation of specific antibody fragments against three model viral antigens: H1N1 influenza virus, Hepatitis B virus (HBV), and Foot-and-mouth disease virus (FMDV) serotype O. The whole FACS screening rounds of the synthetic human antibody library against each viral antigen could be done in one day, and these results show that repeated FACS screening without regeneration of the sorted cells can be a rapid and efficient method to isolate potential antibody candidates in case of urgent requirements.

## Materials and Methods

### Bacterial strain and growth conditions

The bacterial strains and plasmids used in this study are listed in the Table S1. *E. coli* Jude-1 was used as the main host for gene cloning and library screening. *E. coli* HM130 was used for the production and purification of the isolated antibodies (scFv). The *E. coli* cells were inoculated into Luria-Bertani (LB) medium (10 g/L of tryptone, 5 g/L of yeast extract, and 10 g/L of NaCl) containing 2% (w/v) glucose. After an overnight cultivation at 37°C and 200 rpm, the cells were transferred into 100 mL of fresh LB medium without glucose in a 500-mL flask and incubated at 37°C and 200 rpm. When the cell density (OD_600_) reached 0.6, the cells were induced with 1 mM isopropyl-β-d-thiogalactopyranoside (IPTG) and were further cultivated at 25°C at 200 rpm for 4 h. The cells were harvested by centrifugation at 6,000 rpm for 10 min at 4°C for further analysis. In all the cultivations, ampicillin (50 µg/mL) was used as the sole antibiotic.

### FACS screening

For the FACS screening of a human synthetic antibody (scFv) library, three fluorescent antigen probes were chemically synthesized: (i) FITC-CRDNWHGSNRPW as an N1 epitope of H1N1 influenza virus [13]; (ii) FITC-NSTTFHQALLDPRVRGLYFPAGG as a PreS2 epitope of HBV [14]; and (iii) FITC-PVTNVRGDLQVLAQK as a VP1 epitope of FMDV [15]. One aliquot (100 µL) of the library stocks frozen at −80°C was thawed and inoculated into 100 mL of LB medium containing 2% (w/v) glucose and ampicillin. After cultivation under the condition described above, the cells were harvested by centrifuging for 10 min at 6,000 rpm and 4°C. For efficient labeling with fluorescent probes, cell pellets were resuspended in 5× Tris-KCl buffer (150 mM Tris-HCl at pH 7.4 and 750 mM KCl), which dramatically increased the permeability of the *E. coli* outer membrane and allowed the fluorescent antigen probes to permeate into the periplasm [9], [16]. The resuspended cells were incubated with 5 µM of antigen peptides (N1, PreS2, or VP1 epitope) conjugated with FITC for 1 h at 4°C. The cells were then washed twice with the same buffer (5× Tris-KCl), and the fluorescent probe-labeled cells were sorted using a high-speed flow cytometer (Moflo XDP, Beckman Coulter, Miami, FL). In FACS sorting, the cells were selected on the basis of high fluorescence intensity detection through a 530/40 band-pass filter for obtaining the FITC emission spectrum. “Purify mode” was used as the sorting mode, which sorts only those drops that contain positive cells. All the *E. coli* cells sorted in each round of screening were immediately reused for the next round of FACS sorting without regeneration. The sorting was repeated until the highly fluorescent population was fully enriched. To obtain the required sample volume, 500 µL of sheath buffer was added to the sorted samples during each round. After the final sorting, the scFv genes were amplified from the sorted cells by performing PCR with primers, Assembly-F and Assembly-R. After digestion with *Sfi*I, the amplified scFv genes were cloned into pMoPac16 and transformed into *E. coli* Jude-1 for the further analysis of the single clones.

For enrichment of cells producing FlAsH tag (FLNCCPGCCMEP) from non-specific cells, cells harboring pMoPac16-MBP were diluted with non-specific cells harboring pMoPac16 at 1∶10000 ratio. After labeling with with 25 µM of FlAsH-EDT_2_ (Invitrogen), the mixed cells were applied to FACS and the fluorescent cells were sorted by the repeated FACS screening as described above.

### Purification of the antibody fragment

For the production of the isolated antibody fragments, the plasmids recovered from the isolated clones were introduced into *E. coli* strain HM130. After cultivation in 500 mL of LB medium, the cells were harvested by centrifugation for 10 min at 6,000 rpm and 4°C. The cells were then washed twice with PBS and resuspended in the same buffer. Crude extracts of the cells were prepared by sonication (20 min at 50% pulse and 20% amplitude), and the extracts were centrifuged for 10 min at 10,000 rpm and 4°C to yield soluble lysates. The soluble lysates were filtered through a 0.45-µm syringe filter, and the soluble lysates were poured into Poly-prep chromatography columns (Bio-Rad, Hercules, CA) filled with Talon metal-affinity resin (Clontech, Mountain View, CA). The resin was washed twice with 10 mL of washing buffer, and the 6-His tag fused to scFv was eluted using 2 mL of the elution buffer. The purified scFvs were used for ELISA analysis.

### Preparation of Glutathione S-transferase (GST)-fused antigens

For the preparation of GST-fused antigens (N1 of H1N1, PreS2 of HBV, and VP1 of FMDV), three vectors were constructed. The primers used in the construction of the GST-fused antigens are listed in the Table S2. The plasmid pGEX-4T-1 containing the GST gene was used as the template, and the N1 epitope sequence (CRDNWHGSNRPW) of the H1N1 influenza virus [13] was fused to the C-terminus of GST by performing PCR with the primer sets GST-F and GSTN1-R. For the synthesis of the PreS2 epitope sequence (NSTTFHQALLDPRVRGLYFPAGG) of HBV [14] and the VP1 epitope sequence (PVTNVRGDLQVLAQK) of FMDV [15], the same PCR strategy was used, except that the reverse primers—GSTPreS2-R and GSTVP1-R—were used for synthesizing the PreS2 and VP1 epitope genes, respectively. The PCR products containing the GST-fused N1 sequence, PreS2 sequence, or VP1 epitope sequence were digested using *Nde*I and *Hin*dIII, and then were cloned into pMoPac1 to yield pMoPac1-GST-N1, pMoPac1-GST-PreS2, or pMoPac1-GST-VP1, respectively.

*E. coli* Jude-1 harboring pMoPac1-GST-N1, pMoPac1-GST-PreS2, or pMoPac1-GST-VP1 were cultivated in 500 mL of LB medium in a 2-L shaking flask at 37°C at 200 rpm. When the cell density (OD_600_) reached 0.6, the cells were induced with 1 mM of IPTG and were further cultivated at 37°C at 200 rpm for 6 h. The cells were harvested by centrifugation at 6,000 rpm for 10 min at 4°C. The cells were then resuspended in 50 mL of PBS and were lysed by sonication (20 min at 50% pulse and 20% amplitude). After filtration with a 0.45-µm syringe filter (Sartorius Stedim Biotech, Goettingen, Germany) to remove the residual insoluble debris, the GST-fused antigens were purified by using Glutathione Sepharose resin (GE Healthcare Biosciences AB, Uppsala, Sweden) as per the method specified by the manufacturer.

### Enzyme-linked immunosorbent assay (ELISA)

The GST-fused antigens were mixed with 0.05 M carbonate-bicarbonate coating buffer (pH 9.6) to a final concentration of 2 µM. The antigen solution (100 µL) was loaded onto 96-well ELISA plate, which was then incubated for 2 h at 37°C. Subsequently, each well was washed four times with PBS-T (135 mM NaCl, 2.7 mM KCl, 4.3 mM Na_2_HPO_4_, 1.4 mM KH_2_PO_4_, and 0.5% Tween-20 at pH 7.2) and filled with 200 µL of 5% BSA solution, and the plate was incubated for 1 h at 37°C. After washing with PBS-T four times, each scFv sample (soluble lysate or purified sample) was loaded on to the plate, and the plate was incubated overnight at 4°C. Each well was washed four times with PBS-T, after which 1∶5,000-diluted monoclonal Anti-His antibody conjugated to horseradish peroxidase (HRP) (Sigma-Aldrich, ST. Louis, MO) was added, and the plates were incubated for 1 h at 37°C. Finally, the wells were washed with PBS-T, and tetramethylbenzidien (TMB) was added for the colorimetric detection of the bound scFv clones. The reaction was arrested by adding 2 M of H_2_SO_4_ stop solution. The absorbance was measured at 450 nm by using a TECAN Infinite M200 Pro ELISA plate reader (Tecan Group Ltd., Männedorf, Switzerland). To confirm binding activity of scFv against inactivated whole FMDV, an FMDV serotype O kit (PrioCHECK FMDV type O, Prionics, Switzerland) was used; ELISA was performed in the same manner with the exception of the antigen-coating step.

### Surface plasmon resonance (SPR)

For the immobilization onto CM5 chip, all antigens were activated via thiol labeling kit (GE Healthcare, Buckinghamshire, UK). A 0.5 mg of antigens in 0.5 mL were prepared in morpholinoethanesulfonic acid (MES) buffer. Then, 0.25 mL of 2-(2-pyridinyldithio)ethaneamine (PDEA) (15 mg/mL) in 0.1 M MES buffer was added. After adding 25 µL of 0.4 M 1-ethyl-3-(3-dimethylaminopropyl)-carbodiimide (EDC), the mixtures were incubated at 25°C for 10 min. The buffers were exchanged to PBS via dialysis after the incubation. A Biacore 3000 (GE Healthcare) and CM5 chip (GE Healthcare) was used in all SPR analysis. SPR experiments were carried out at a flow rate of 10 µL/min using PBS as running buffer. For the activation of the surface of CM5 chip, 0.2 M EDC and 0.5 M N-hydroxysuccinimide (NHS) solution was injected for 2 min. And 40 mM cystamine solution was injected for 3 min, and then, 0.1 M dithioerythritol (DTE) in 0.1 sodium borate solution was injected for 3 min. After the activation of the chip surfaces, 100 µg/mL of activated antigens were injected for 7 min. For deactivation of excess reactive groups on the chip surface, 20 mM PDEA and 1 M NaCl solution in 0.1 M sodium acetate was injected for 4 min. Finally, various concentrations of isolated scFvs were injected for 5 min and the binding signal was detected. The K_D_ values of scFvs were evaluated by measuring K_on_ and K_off_ in BIA evaluation software.

### Other analytical methods

Protein samples were analyzed by performing electrophoresis on a 12% (w/v) SDS-polyacrylamide gel electrophoresis (SDS-PAGE) gel. For the immunodetection of the His-tag-fused scFv proteins, a monoclonal anti-His antibody conjugated to horseradish peroxidase (HRP) (Sigma-Aldrich) was used. An ECL kit (Amersham ECL Prime Western Blotting Detection Reagent, GE Healthcare) was used for signal detection.

## Results

### The proof of repeated FACS screening strategy

**Figure 2:**
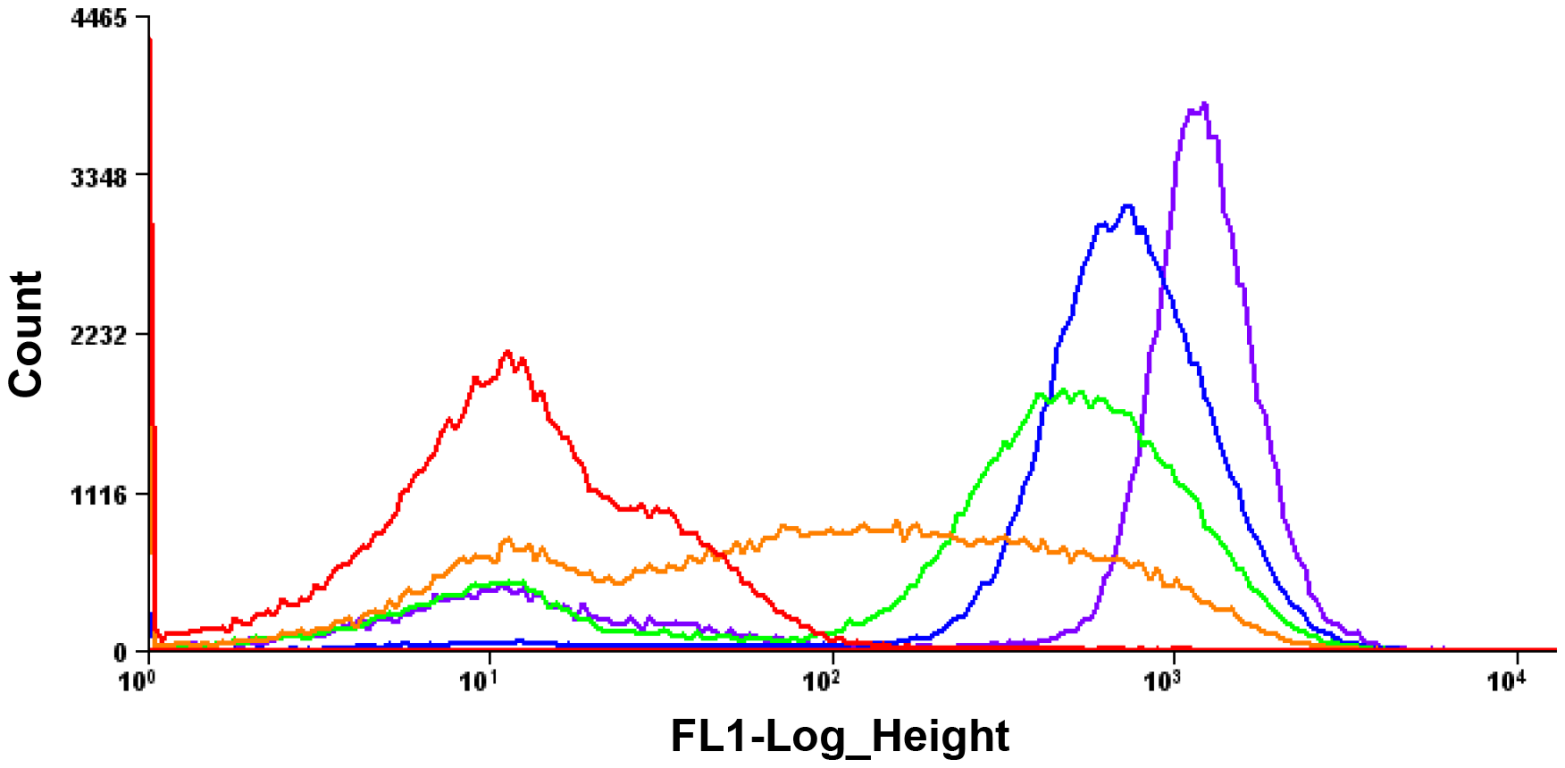
Enrichment of probe-specific clones by the repeated FACS screening. The FlAsH-EDT_2_ labelled cells producing FlAsH-tagged MBP were enriched from overwhelming amount of non-fluorescent cells (1∶10^4^ dilution) by repeated FACS screening. The histograms of original library, 1^st^ round, 2^nd^ round, 3^rd^ round, and 4^th^ round sorted cells are represented by red, orange, green, blue, and purple curves, respectively.

To prove the concept of our strategy (repeated FACS screening without regeneration) (Fig. 1), we first conducted the enrichment of probe-specific cells from mixture with non-specific cells. In this enrichment experiment, FlAsH-EDT_2_ which can interact with FlAsH tag (FLNCCPGCCMEP) and give fluorescent signal [17] was used as probe for cell labeling. Cells producing FlAsH-tag fused maltose binding protein (MBP) were mixed with cells producing no-tag protein at 1∶10000 dilution ratio, and the enrichment of positive cells were performed by repeated FACS screening strategy. From the mixture, the fluorescent cells (top ∼1.5% of the total population) were sorted and, immediately after the first round sorting, the sorted cells were applied to the next round of sorting by FACS without cell regeneration. As shown in Fig. 2, the fluorescent cells began to be enriched after second round and fully enriched after fourth round sorting. This successful enrichment of probe-specific clones clearly indicate the repeated FACS screening strategy can be used for the rapid screening of antigen-specific antibody from library.

### Design and construction of a synthetic human antibody library

A fully synthetic human antibody (scFv) library with six diversified complementarity-determining regions (CDRs) was constructed. The detailed procedures for synthetic library construction are described in the Figure S1. Briefly, by following the Kabat definition [18] and using the database of human germline genes (http://www.bioinf.org.uk/abysis), the single chain variable fragment (scFv) was designed to have eight framework regions (FRs) and six CDRs. Regarding the frequency of usage and expression in *E. coli*, the DP-47 and DPK 22 human germline genes were chosen for the framework region of VH and VL, respectively. For the diversity of each CDR loop, except CDR H3, degenerate oligonucleotides were designed on the basis of the frequency of amino acids for each site in the human germline gene database (Table S3). In the case of CDR H3, much greater diversity in amino acid composition and length was introduced, unlike that in the case of the other CDRs, by using 13 sets of degenerate oligonucleotides with an NNK random sequence and 13 different lengths (10–22 amino acids) (Fig. S1). Each VH and VL gene was assembled by PCR and, to eliminate the clones containing stop codons or frameshifts in the VH or VL gene, each PCR product of VH and VL was cloned into a β-lactamase fusion system. In the selection system, the *E. coli* clones containing in-frame genes of the each chain produced the functional β-lactamase fusion proteins in the periplasm and were selected using ampicillin-containing agar plates [19], [20]. After selection, the in-frame VH and VL genes were rescued, and the gene sequences were determined by sequencing. Through sequencing experiments, we found that all the clones contained in-frame codons, and no stop codons were observed within the coding genes (data not shown). The rescued VH genes and VL genes were linked for a full antibody fragment (scFv) format and the in-frame scFv genes were also selected in the β-lactamase fusion system. From the selected clones, all in-frame scFv genes were rescued and cloned into pMoPac16 for constructing a synthetic human antibody (scFv) library. From the library, 20 clones were randomly chosen, and their sequences were analyzed, and it was clearly confirmed that all 20 clones contained various sequences in the CDRs (Fig. S2). Also the randomly selected clones showed good levels of scFv gene expression in *E. coli* (Fig. S3). The size of the final constructed library was about 3.3×10^6^, which is sufficient for the initial isolation of antibody candidates against the target antigens. With this synthetic library, we conducted the isolation of antibody candidates against three antigens: (i) N1 antigenic epitope of H1N1 influenza virus; (ii) PreS2 antigenic epitope of HBV; and (iii) VP1 antigenic epitope of FMDV.

### Isolation of antibody fragment against the N1 epitope of the H1N1 influenza virus

**Figure 3:**
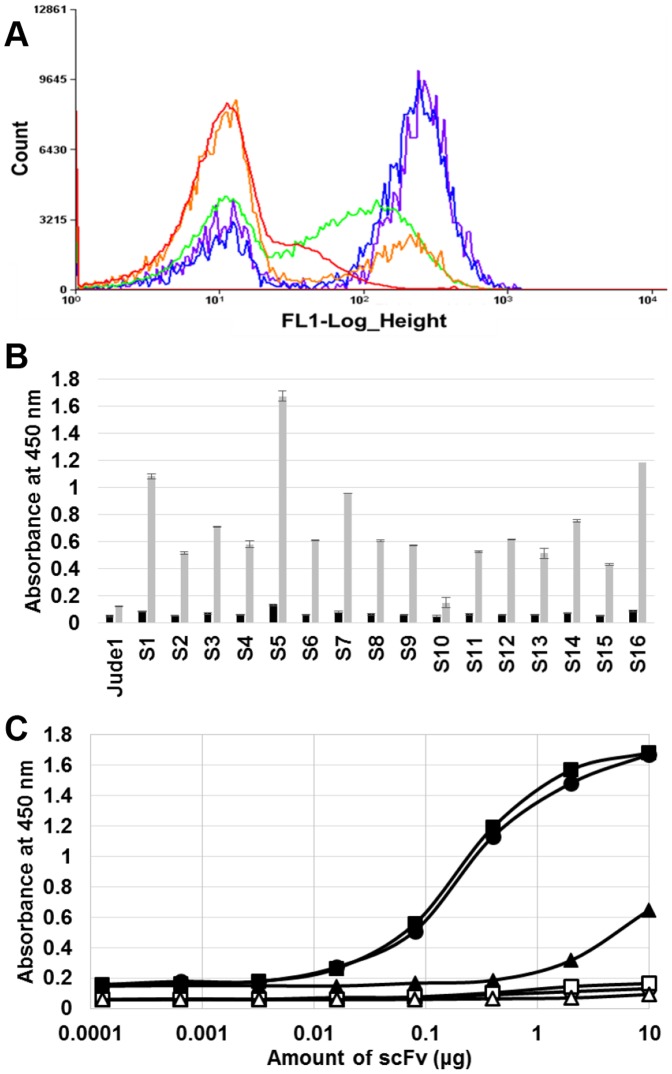
Isolation of antibody against N1 epitopes of H1N1. (A) FACS enrichment of cells. The histograms of original library, 1^st^ round, 2^nd^ round, 3^rd^ round, and 4^th^ round sorted cells are represented by red, orange, green, blue, and purple curves, respectively. The 5^th^ round sorted cells were used for regeneration of scFv genes by PCR, and its histogram is not shown here. (B) ELISA with the soluble lysates of the selected clones. Black bar indicate signals from BSA coated wells, and grey bars indicate signals from GST-fused N1 antigen coated wells. (C) ELISA analysis with the purified scFvs. Symbols: circle, square and triangle represent the purified antibody from clones S1, S5 and S16, respectively. The closed and open symbols represent the coating of GST-fused N1 antigen and BSA on 96-well plates, respectively.

In order to isolate the N1 epitope-specific antibody (scFv), the synthetic library was screened as shown in the Figure 1. After cultivation of library, cells were harvested and treated with 5× Tris-KCl buffer to improve the permeability of the outer membrane and to increase the labeling efficiency of the cells for fluorescent probes [9], [17]. Then, cells were mixed with 5 µM of N1 antigenic peptide conjugated with FITC, and cell fluorescence was analyzed by FACS. Compared to the negative control (*E. coli* harboring pMoPac16 vector only), the cells from the original library showed a slightly higher, but mostly similar fluorescence intensity (Fig. 3A). From the original library, the most fluorescent cells (top ∼0.28% of the total population) were sorted until the number of sorted events reached 510,434. Immediately after the first round of sorting, the sorted cells were applied to a second round of sorting by FACS, and the most fluorescent population of the sorted cells (top ∼3.06% of total population) were sorted again. After repeating this sorting three more times, the highly fluorescent population became the major population in the final (5^th^) round of sorting. The fluorescence of the sorted cells was clearly observed to be fully separated from that of the original library (Fig. 3A). Finally, 1,561 fluorescent cells were collected and used for analysis of individual clones. The sorting results are summarized in the Table 1.

**Table 1:** Overall results of antibody library screening by repeated FACS sorting.

Round	N1 of H1N1		PreS2 of HBV		VP1 of FMDV	
	Sort count	Sort %	Sort count	Sort %	Sort count	Sort %
**1^st^**	510,434	0.28	1,531,233	5.53	246,139	1.87
**2^nd^**	31,171	3.06	13,271	16.72	11,173	33.29
**3^rd^**	10,025	6.36	2,722	16.87	5,262	87.41
**4^th^**	3,620	49.84	1,412	40.10	-	-
**5^th^**	1,561	46.30	-	-	-	-

After the final screening, the scFv genes of the 1,561 sorted cells were rescued by PCR, and the PCR product was cloned into pMoPac16 for the periplasmic expression of scFvs. After transformation into *E. coli* Jude-1, a total of 16 colonies were randomly picked from the agar plate, and the binding activities of the isolated antibodies were analyzed by performing ELISA. Most clones, except for one (S10), exhibited higher binding activity against the N1 peptide than the non-specific BSA control (Fig. 3B). From the sequencing experiments, we found that among the 16 clones, six clones including S2, S4, S6, S9, S11, and S13, had the same DNA sequences. Two other clones, S8 and S12, were also same clones, while another two clones, S3 and S10, were same. The same clones with the same sequences showed similar levels of binding activity, except for S3 and S10 (Fig. 3B). Among the 16 clones, four, including S1, S5, S7, and S16, showed relatively higher binding activity than the other clones; therefore, we tried purification of scFvs to analyze their specific activities. The four scFv genes were transformed into the *E. coli* strain HM130, which is protease-deficient and suitable for higher scFv production [21]. After cultivation, scFvs of three clones S1, S5, and S7 could be purified with high purity, but we failed to purify scFv of clone S16 (data not shown). The specific activities of three purified scFvs (S1, S5, and S7) against the N1 epitope were analyzed by ELISA and we observed that the binding activities of S1 and S5 were higher than that of S7 and a non-specific BSA control (Fig. 3C). The sequence information of three isolated scFv (S1, S5 and S7) was provided in the Figure S4.

### Isolation of antibody fragments against the PreS2 antigenic epitope of HBV

**Figure 4:**
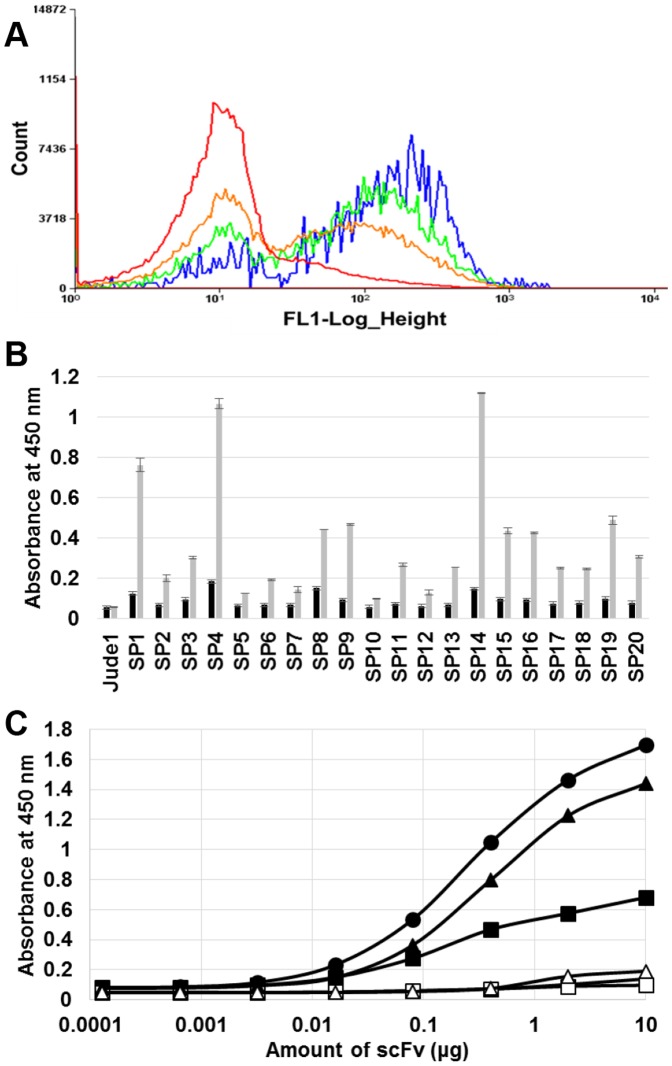
Isolation of antibody against PreS2 epitopes of HBV. (A) FACS enrichment of cells. The histograms of original library, 1^st^ round, 2^nd^ round, and 3^rd^ round sorted cells are represented by red, orange, green, and blue curves, respectively. The 4^th^ round sorted cells were used for regeneration of scFv genes by PCR, and its histogram is not shown here. (B) ELISA analysis with the soluble lysates of the selected clones. Black bar indicate signals from BSA coated wells, and grey bars indicate signals from GST-fused PreS2 antigen coated wells. (C) ELISA analysis with the purified scFvs. Symbols: circle, square and triangle represent the purified antibody from clones SP1, SP4 and SP19, respectively. The closed and open symbols represent the coating of GST-fused PreS2 antigen and BSA on 96-well plates, respectively.

To demonstrate the general use of our strategy for antibody screening, we also isolated a potential antibody against another target, PreS2 of HBV. After cultivation and permeabilization of the library, cells were labeled with PreS2 antigenic peptide conjugated with FITC, and the labeled library cells were sorted by FACS. From the original library, the most fluorescent cells (top ∼5.53% of total population) were sorted until the number of sorted events reached 1,531,233. Then, through repeated sorting without regeneration (three more times), highly fluorescent cells were enriched as a major population, and finally 1,412 cells were collected (Fig. 4A and Table 1). The scFv genes from the final 1,412 sorted cells were regenerated by PCR, cloned into pMoPac16, and then transformed into *E. coli* strain Jude-1. Among the regenerated clones, 20 total colonies from the plate were randomly picked, and their binding activities were analyzed by ELISA. Most clones exhibited good binding activity against antigen PreS2 of HBV, but among the 20 clones, four (SP1, SP4, SP14, and SP19) exhibited relatively higher binding activity than the other clones (Fig. 4B). From the sequencing experiment, it was confirmed that two clones (SP4 and SP14) were identical, and so three clones (SP1, SP4, and SP19) were chosen for further analysis. After cultivation, each antibody was successfully purified (data not shown) and the specific binding activities of purified antibodies were evaluated by ELISA. We observed that the scFvs purified from clones SP1 and SP19 had much higher binding activity than that from the SP4 clones (Fig. 4C). The sequence information of three isolated scFv (SP1, SP4, and SP19) was provided in the Figure S4.

### Isolation of antibody fragments against the VP1 antigenic epitope of FMDV

**Figure 5:**
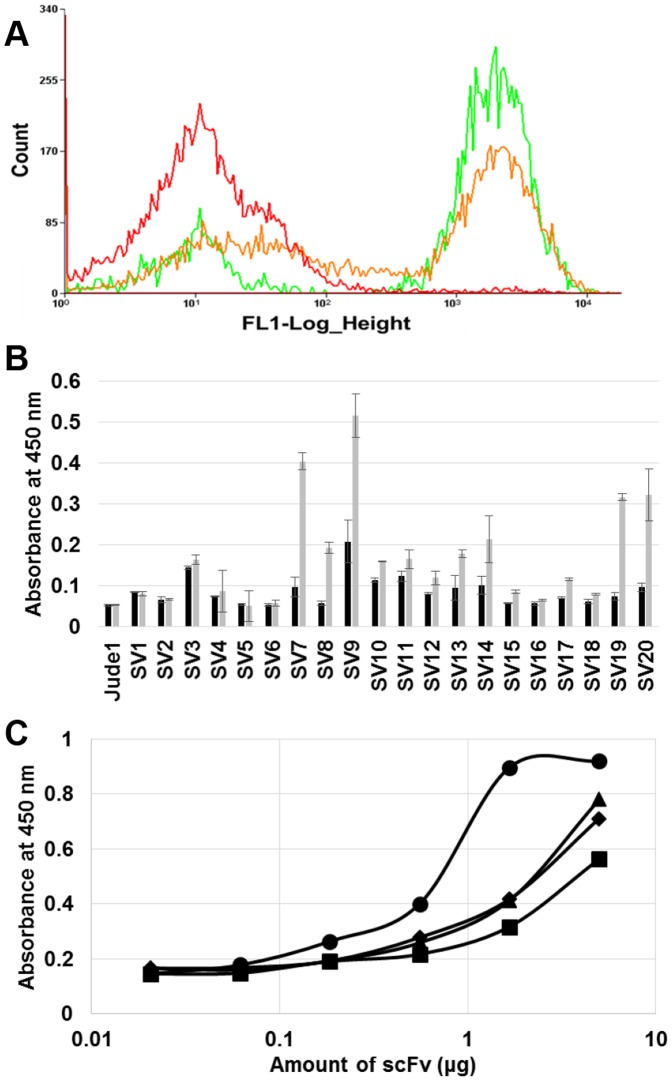
Isolation of antibody against VP1 epitopes of FMDV. (A) The histograms of original library, 1^st^ round, and 2^nd^ round sorted cells are represented by red, orange, and green curves, respectively. The 3^rd^ round sorted cells were used for regeneration of scFv genes by PCR, and its histogram is not shown here. (B) ELISA with the soluble lysates of selected clones. Black bar indicate signals from BSA coated wells, and grey bars indicate signals from GST-fused VP1 antigen coated wells. (C) ELISA analysis with the purified scFvs. Symbols: circle, square, triangle and diamond represent the purified antibody from clones SV7, SV9, SV19, and SV20, respectively. The signals were detected from the wells coated with GST-fused VP1 antigen on 96-well plates.

**Figure 6:**
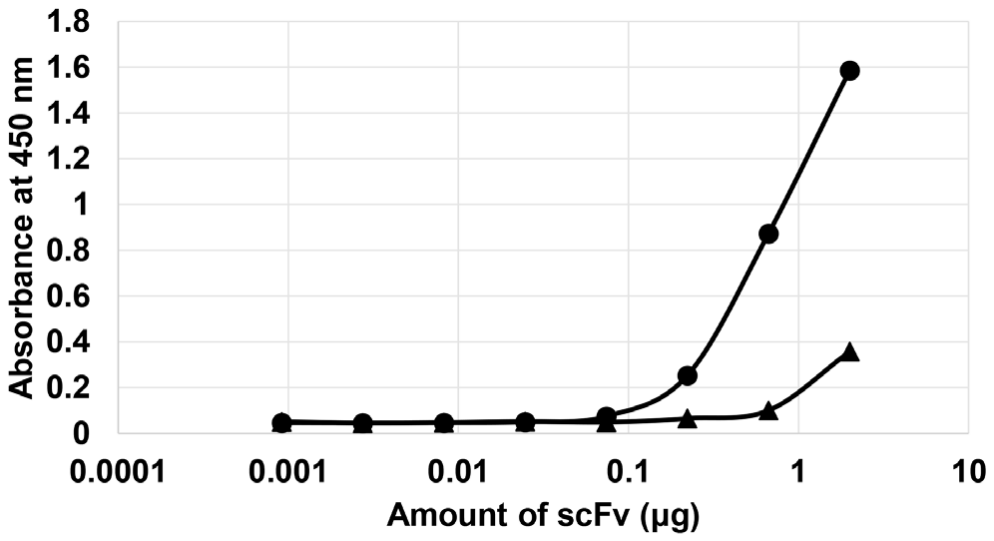
ELISA analysis using FMDV serotype O kit of which wells are coated with inactivated real FMDV. Symbols; Circle and triangle represent SV7 scFv and M18 scFv, respectively.

Foot-and-mouth disease (FMD) is a highly contagious viral disease infecting cloven-hoofed animals, such as cattle and swine, and it has resulted in massive livestock losses around the world. The diagnosis of FMDV at an early-stage of contamination is crucial to the prevention of the contagion. For the development of an immunodiagnostic system, a highly specific antibody is necessary; therefore, we chose the VP1 antigenic epitope of FMDV as another target antigen for the application of the repeated FACS screening system. The synthetic antibody library cells were cultivated and labeled with FITC-conjugated VP1 antigenic peptide, and the labeled library cells were sorted by FACS. From the original library, the most fluorescent cells (top ∼1.87% of total population) were sorted until the number of sorting events reached 246,139. After two more rounds of FACS screening, the highly fluorescent cells were enriched as a major population, and finally 5,262 cells were collected (Fig. 5A and Table 1). The scFv genes from the final 5,262 sorted cells were regenerated by PCR, cloned into pMoPac16, and then transformed into *E. coli* strain Jude-1. From the regenerated cells, we randomly picked 20 clones and obtained four clones (SV7, SV9, SV19, SV20) expressing active scFvs against the VP1 antigenic epitope (Fig. 5B). The four scFvs (SV7, SV9, SV19, SV20) that showed high binding were purified for further analyses. In ELISA, we observed that one scFv (SV7) has much higher binding activity than the others (Fig. 5C). Finally, we also examined the binding activity of the isolated scFv (SV7) against whole (inactivated) FMDV. It was clearly observed that the isolated SV7 antibody fragment exhibited high binding activity against FMDV, while negative control M18 scFv, which can specifically bind to anthrax toxin PA [11], showed a negligible signal (Fig. 6). The sequence information of four isolated scFv (SV7, SV9, SV19 and SV20) was provided in the Figure S4.

### Binding affinity of the isolated antibody

The binding activity (K_D_) of isolated antibody against each antigen were also determined by SPR analysis. Each antigen was immobilized on CM5 chip and the purified antibodies were loaded and binding affinities were analyzed. The binding affinities (K_D_s) of isolated antibodies, S5 scFv against N1 epitope, SP1 scFv against PreS2, and SV7 scFv against were 11 nM, 11.5 nM and 54.9 nM, respectively (Fig. S5), which were similar to those typically obtained using hybridoma technology and other antibody screening strategies including phage display *etc*. In SPR analysis, the affinity can be overestimated due to the avidity effect of dimerized or multimerized antibodies. To check the presence of dimeric antibodies in purified samples, all purified antibodies were analyzed by size exclusion column chromatography and by SDS-PAGE analysis in non-reducing and reducing conditions. In all experiments, we clearly confirmed the purified samples were present mainly as a monomeric form (Fig. S6 and Fig. S7) and so the possible avidity effect in SPR analysis could be excluded. And the specificity of the isolated scFvs were also confirmed further by western blot on complex protein mixtures containing wildtype GST or antigen-fused GST. As shown in the Figure S8, each scFv could bind specifically to its own viral antigen-fused GST in the soluble lysate of *E. coli* host. Taken all these data, we concluded that, in our screening, each antibody could be isolated not by non-specific binding of antibody on the cell surface (stickiness) but by specific binding of antibody to antigen probe, and this means that our strategy is valid for the rapid isolation of antibody from library.

## Discussion

Screening libraries constructed by animal immunization (mainly mouse) with antigens has several benefits, but mouse antibodies give rise to a human anti-murine antibody response (HAMA) [22]. To reduce the undesired immunogenicity of the isolated mouse antibodies, “humanizing” procedures are commonly required, in which the non-human CDRs are conjugated into a given human sequence [23]. However, the exchange of the mouse framework with the human framework does not always guarantee similar affinity, and often leads to less immunogenicity; therefore, it is not suitable for the rapid isolation of effective antibodies. Instead of an immune antibody library, non-immune synthetic antibody libraries have been developed that are generally constructed in human antibody frameworks [24], [25]. In the synthetic library, the quality and diversity are of particular importance in the isolation of the antibody; therefore, the six complementarity-determining regions are fully randomized and inserted into a single human antibody framework. For the efficient and soluble production of antibodies in a bacterial host, as well as for less immunogenicity in the human body, the choice of framework in library construction also needs to be considered seriously, and therefore, the isolated clones from the synthetic library are likely to have favorable properties for production and further therapeutic applications. Within the human genome, it is known that 51 V_H_, 30 V_λ_, and 40 V_κ_ gene segments exist. Although all gene segments can produce a functional antibody, the level of gene expression and the stability of the antibody are very different [24]. In our synthetic library, the DP-47 and DPK 22 human germline genes, which are highly prevalent in human antibodies and are known to be well expressed in *E. coli* [26]–[29], were chosen for the framework region of VH and VL, respectively. The resulting antibody library showed diversity size of 3.3×10^6^. We know that this library size is relatively small compared to diversity of natural human antibody repertoire and, the construction of much bigger library (>10^9^) is needed to increase the probability for the generation of antibodies against various antigens. However, although the size of library is not large, the quality of our library was enough to be used for the isolation of antigen-specific antibody. To reduce the incidence of frame shifts and stop codons during library construction, the libraries were subjected to in-frame selection by fusion with β-lactamase and ampicillin resistance selection. The quality of the library was validated by sequencing clones that were randomly selected from the library and by analysis of antibody production in *E. coli* (Fig. S2). Most clones selected randomly from the constructed library and isolated clones by screening exhibited good expression levels with high solubility in *E. coli*; therefore, we concluded that the high-quality human antibody library with high diversity in six CDRs was successfully synthesized and was suitable for the rapid isolation of antigen-specific antibodies.

For the isolation of highly potential clones from these huge libraries, large microbial libraries need to be screened through multiple rounds of sorting due to the contamination of non-specific false-positive clones; however, in most strategies, this regeneration step takes a long time, which makes it difficult to rapidly isolate potential antibodies from a combinatorial library [30]. Our strategy, however, does not include time-limiting regeneration steps for the sorted population. The sorted population is used immediately for the next round of sorting, and this re-sorting process is repeated until the highly fluorescent population becomes separated from the negative population. Without this regeneration step, highly fluorescent cells that produce potential antibody candidates could be collected in four hours through repeated sorting. For this sorting, we used high-speed FACS sorter which can screen up to 70,000 cells per sec and, with this sorting rate, the first round to sort 0.5 to 1.5 million cells from library took approximately 2 h and all repeated sorting (4–5 times) could be completed in 4 hours. In the initial round of sorting, false-positive clones, where fluorescent antigen probes are non-specifically bound to antibodies on cells, can be found; however, those clones can be removed during the repeated sorting due to the fact that the binding of these antigen probes is not strong or cannot be maintained over repeated sorting for four hours. With this strategy, the overall procedure from cell cultivation to final round sorting could be completed in one day, with much time saved, unlike that in the previous strategies. In addition, we need to consider the effect of differing cell growth on the sorted cell population in the next round of sorting. Stressful conditions during the FACS screening process and antibody expression can severely affect cell viability [12]. Therefore, during overnight cultivation for regeneration, the more viable cells tend to overgrow and become the major population simply because of their viability, and not because of their binding activity. The use of this unfavorable population in the next round of screening makes it difficult to isolate the positive clones. However, by eliminating the need for regeneration, our strategy can minimize the risk of overgrowth of false-positive clones, as well as losing positive candidates during the sorting process. Consequently, antibody candidates can be rapidly isolated in one day, as we successfully demonstrated with three antigen models (N1 antigenic epitope of H1N1 influenza virus, PreS2 antigenic epitope of HBV, and VP1 antigenic epitope of FMDV), where the isolated antibodies exhibited highly specific and strong activity against their antigen targets.

Although we could get successful screening results for three examined antigens, it does not mean that the use of our screening strategy guarantee the successful screening of antibody against various antigens. As one limitation, we can consider the variability of the performance of FACS screening in respect of individual antigen. Due to the different properties (stickiness, size and permeability) of individual antigen probes, cells can be non-specifically labeled with probes and, unwanted screening results (isolation of non-specific and low-affinity antibody) can be obtained. To minimize the non-specific labeling of probe, we may need to modify the labeling conditions and display system according to the properties of probes. For example, in current display system, big size (>10 kDa) probe cannot be used for screening because it cannot enter to periplasm of cell, and so our strategy is not suitable for antibody screening against big size antigens including whole virus. To accommodate big size probes, we can suggest the use of APEx system [11], [31] instead of current periplasmic expression system. In APEx system, outer membrane can be partially removed by spheroplasting treatment which allows the entrance of big size antigens to periplasm. With APEx system, it was already demonstrated the antibody can interact with antigen as large as 250 kDa in periplasm of *E. coli* [11]. Our strategy can be easily combined with APEx system. Using APEx system, cells can be labeled with big size antigens, and the positively labeled cells can be isolated by our repeated sorting. Like this, the display system and labeling conditions can be modified if necessary, and the isolation of positive clones can be done by repeated sorting strategy.

In conclusion, we developed a new strategy for isolating antigen-specific antibodies in *E. coli* by simply repeating FACS using a high-speed cell sorter. A fully synthetic human antibody library with six diversified CDRs was constructed in *E. coli* [32], and with repeated sorting, potential antibody candidates against three viral antigens (N1 of H1N1 influenza virus, PreS2 of HBV, and VP1 of FMDV) could be successfully isolated in one day. The isolated antibody candidates were then easily purified, and their high activities against each antigen were confirmed by performing ELISA. In the case of FMDV VP1, the final isolated antibody candidate (SV7) was also revealed to have high affinity against not only the VP1 antigenic peptide probe, but also the entire FMDV. Therefore, this study has shown that repeated FACS screening without regeneration of sorted cells can be an alternative strategy to isolate potential antibody candidates for therapeutic or diagnostic use in emergencies, and our screening strategy is expected to be used in situations requiring a rapid response to spreading disease [33].

## Supporting Information

Figure S1 **Schematic diagram of oligonucleotides assembly for construction of variable heavy chain (VH) and variable light chain (VL) libraries.** The number on each fragment indicates the number of primer used for PCR.
(TIF)

Figure S2 **Amino acid sequence of 20 clones randomly selected from synthetic antibody library.**
(TIF)

Figure S3 **Western blot analysis of randomly picked 10 clones of synthetic antibody library.** T indicates total lysates, and S indicates soluble lysates.
(TIF)

Figure S4 **Amino acid sequence of the isolated antibody against three antigens.** S1, S5 and S16 scFvs are against N1 epitope of H1N1; SP1, SP4, and SP19 scFv are against PreS2 epitope of HBV; SV7, SV9, SV19 and SV20 scFvs are against VP1 of FMDV.
(TIF)

Figure S5 **Surface Plasmon Resonance analysis for calculation of K_D_ values of isolated antibodies.** A: Anti-N1 S5 scFv, B: anti-PreS2 SP1 scFv, C: anti-VP1 SV7 scFv. The different concentrations of antibody samples are shown with each curve.
(TIF)

Figure S6 **Size exclusion chromatography for purified scFvs which were used in SPR analysis.** A: Anti-N1 S5 scFv, B: anti-PreS2 SP1 scFv, C: anti-VP1 SV7 scFv. D: Standards (Ovalbumin (43 kDa), M18 scFv [11] (27 kDa)). The curve indicates detection of proteins in the chromatography. (X-axis: volume, Y-axis: UV detection (mAU))
(TIF)

Figure S7 **SDS-PAGE and Western blot analysis of purified scFvs which were used for SPR analysis in non-reducing and reducing conditions.** A: SDS-PAGE analysis, B: Western blot analysis. (N indicates non-reducing condition and R indicates reducing condition.)
(TIF)

Figure S8 **Western blot on complex protein mixture to confirm specificity of isolate scFvs.** A: SDS-PAGE and B: Western blot analysis against cell extracts containing wild type GST (lanes G) or antigen fused GST (lane N, P and V). (N, N1 of H1N1 influenza virus; P, PreS2 of HPV; V, VP1 of FMDV). For western blot analysis, the cell extracts were labeled with S5, SP1, or SV7 scFv, then detected with anti-His HRP antibody. Closed arrowhead in lanes N, P, and V indicate protein bands of viral antigenic peptide fused GST. Open arrowheads in lanes G indicate protein bands of wild type GST. Arrows in lanes N and P indicate the possible degraded forms of antigen-fused GST.
(TIF)

Table S1 **Bacterial strains and plasmids used in this study.**
(DOCX)

Table S2 **Primers used for construction of GST-fused antigens, sFGFP, MBP.**
(DOCX)

Table S3 **Primers used for construction of synthetic antibody library.**
(DOCX)
