# Supplementary figures and images for: Rapid Isolation of Antibody from a Synthetic Human Antibody Library by Repeated Fluorescence-Activated Cell Sorting (FACS)
Source: PLoS One. 2014 Oct 10;9(10):e108225. doi: 10.1371/journal.pone.0108225 (PMC4193741; doi:10.1371/journal.pone.0108225)

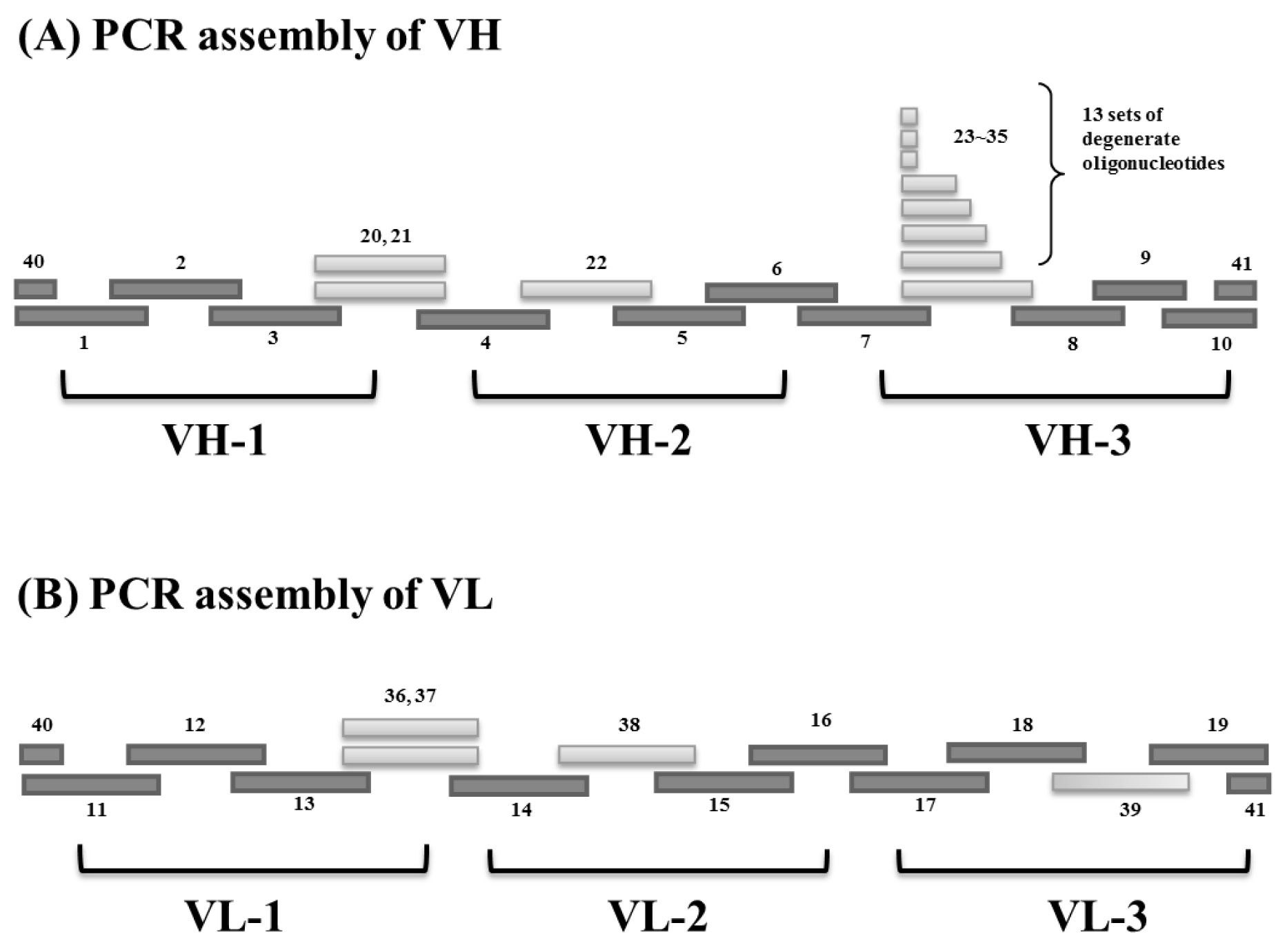

Supplement: Figure S1 — Schematic diagram of oligonucleotides assembly for construction of variable heavy chain (VH) and variable light chain (VL) libraries. The number on each fragment indicates the number of primer used for PCR. (TIF) [file pone.0108225.s001.tif]

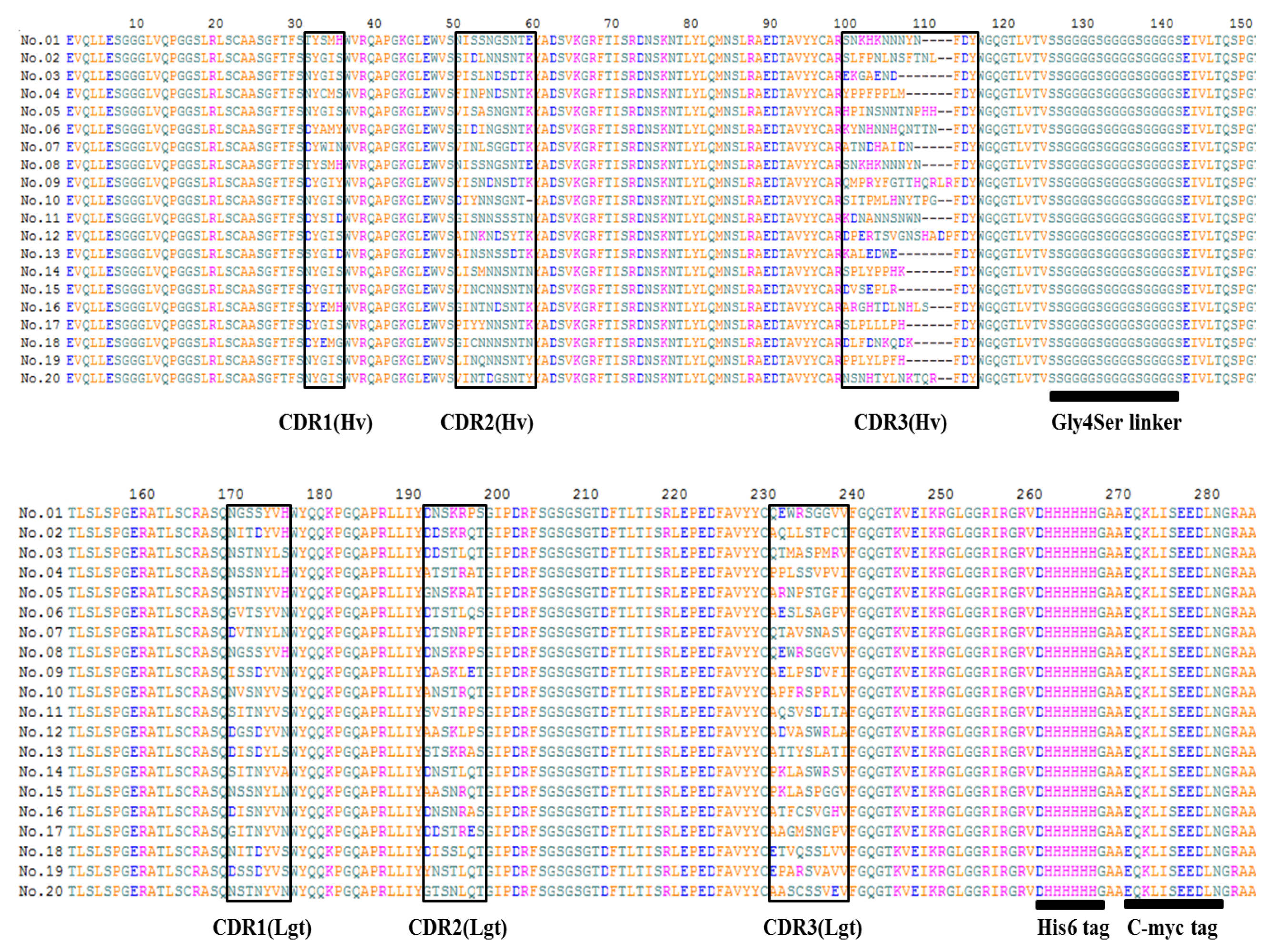

Supplement: Figure S2 — Amino acid sequence of 20 clones randomly selected from synthetic antibody library. (TIF) [file pone.0108225.s002.tif]

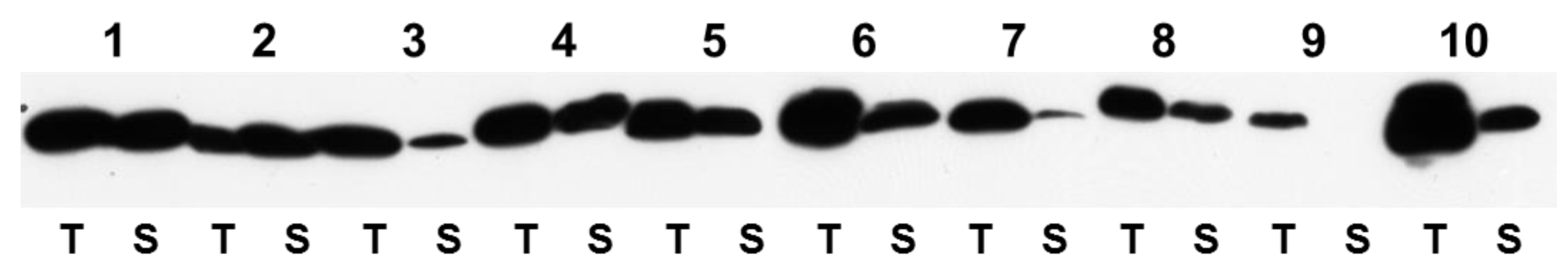

Supplement: Figure S3 — Western blot analysis of randomly picked 10 clones of synthetic antibody library. T indicates total lysates, and S indicates soluble lysates. (TIF) [file pone.0108225.s003.tif]

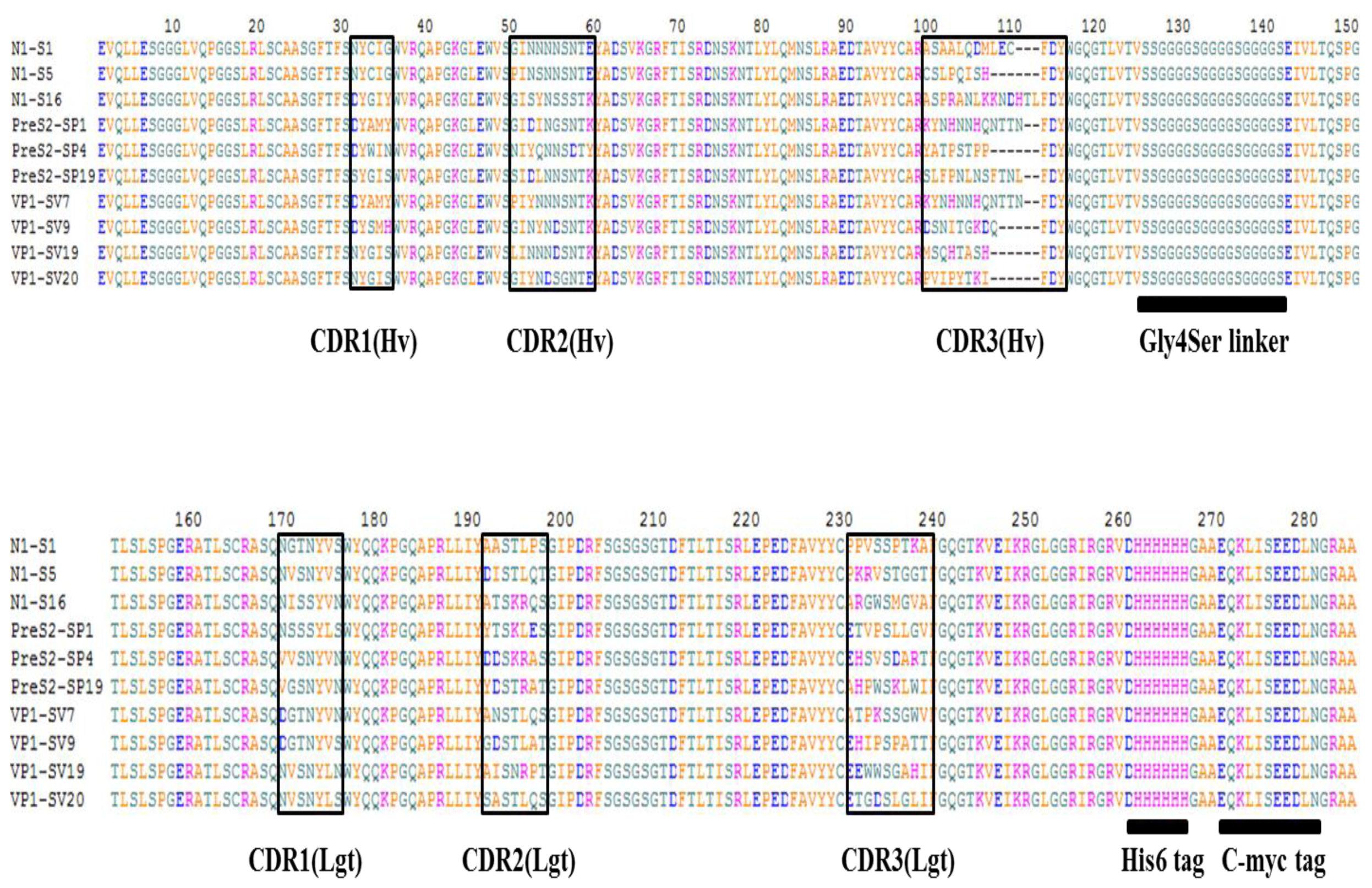

Supplement: Figure S4 — Amino acid sequence of the isolated antibody against three antigens. S1, S5 and S16 scFvs are against N1 epitope of H1N1; SP1, SP4, and SP19 scFv are against PreS2 epitope of HBV; SV7, SV9, SV19 and SV20 scFvs are against VP1 of FMDV. (TIF) [file pone.0108225.s004.tif]

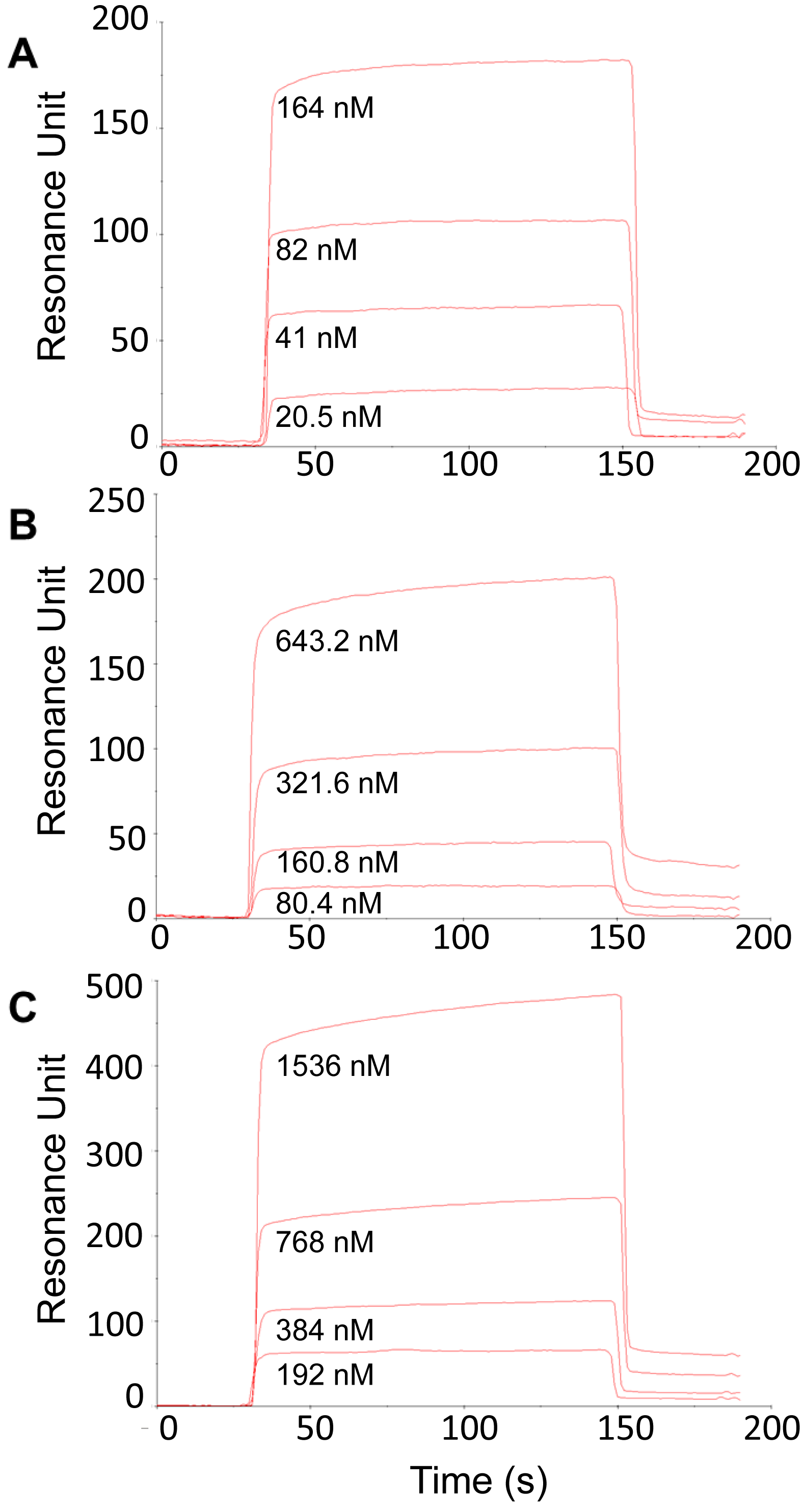

Supplement: Figure S5 — Surface Plasmon Resonance analysis for calculation of KD values of isolated antibodies. A: Anti-N1 S5 scFv, B: anti-PreS2 SP1 scFv, C: anti-VP1 SV7 scFv. The different concentrations of antibody samples are shown with each curve. (TIF) [file pone.0108225.s005.tif]

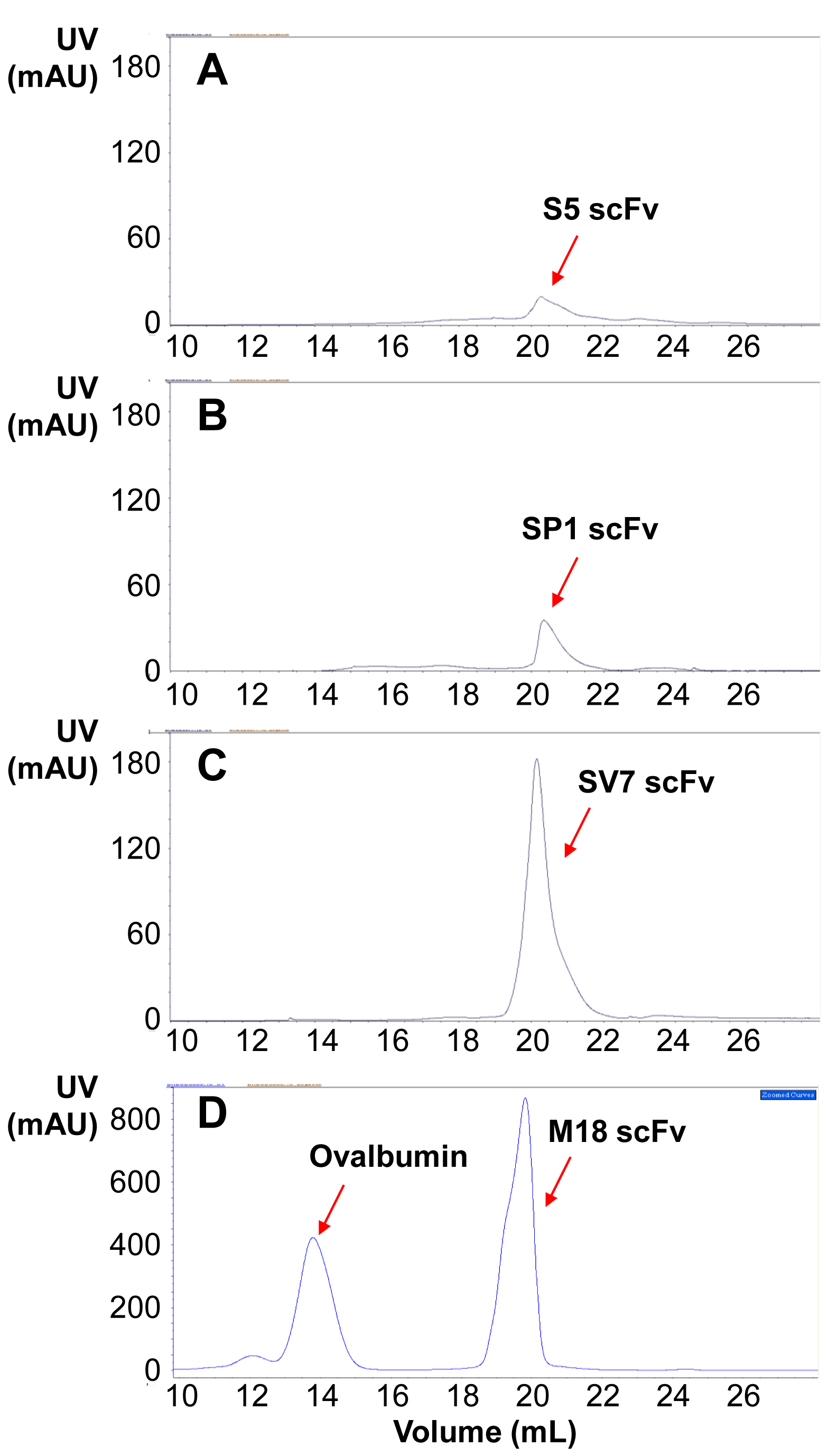

Supplement: Figure S6 — Size exclusion chromatography for purified scFvs which were used in SPR analysis. A: Anti-N1 S5 scFv, B: anti-PreS2 SP1 scFv, C: anti-VP1 SV7 scFv. D: Standards (Ovalbumin (43 kDa), M18 scFv [11] (27 kDa)). The curve indicates detection of proteins in the chromatography. (X-axis: volume, Y-axis: UV detection (mAU)) (TIF) [file pone.0108225.s006.tif]

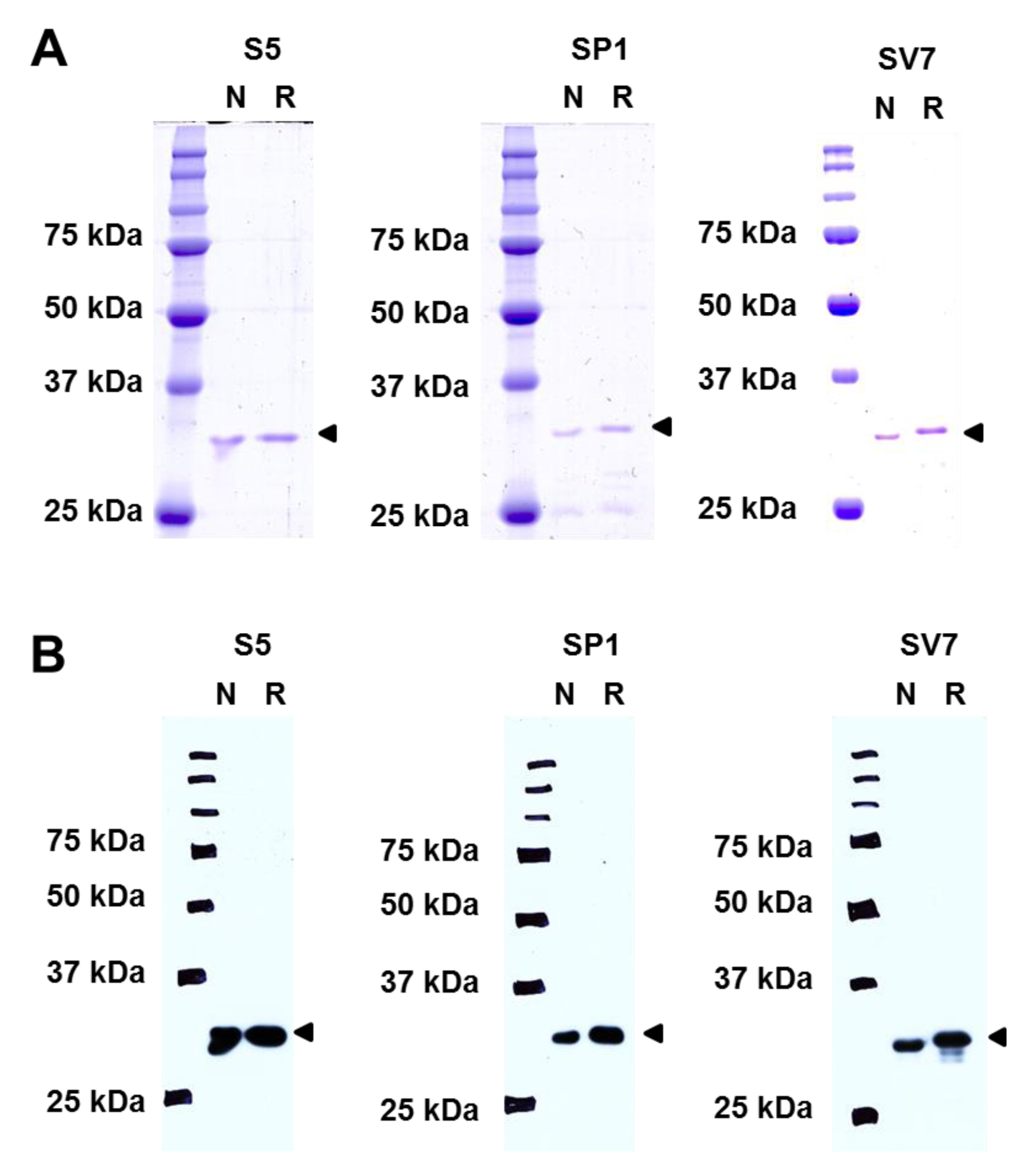

Supplement: Figure S7 — SDS-PAGE and Western blot analysis of purified scFvs which were used for SPR analysis in non-reducing and reducing conditions. A: SDS-PAGE analysis, B: Western blot analysis. (N indicates non-reducing condition and R indicates reducing condition.) (TIF) [file pone.0108225.s007.tif]

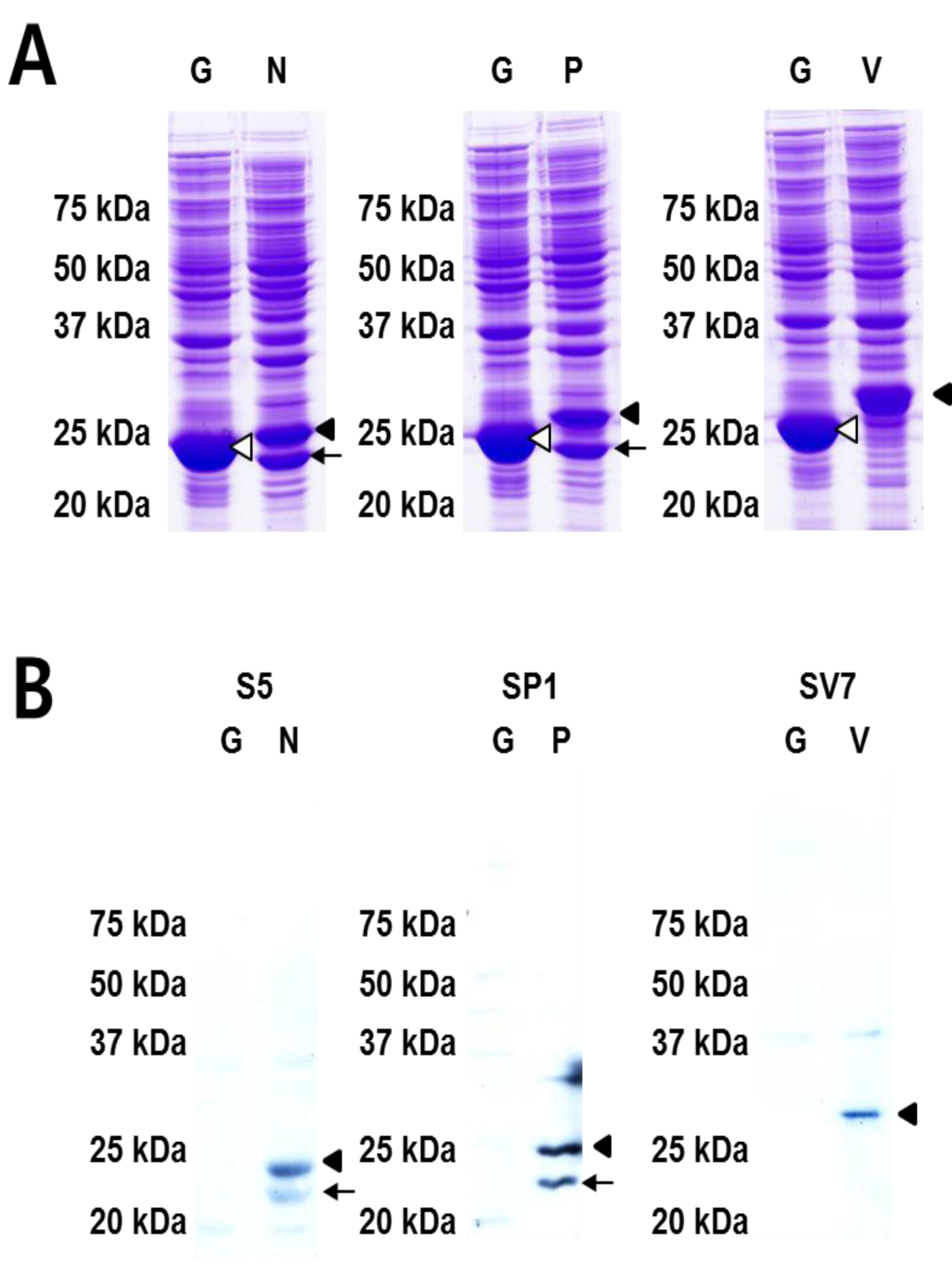

Supplement: Figure S8 — Western blot on complex protein mixture to confirm specificity of isolate scFvs. A: SDS-PAGE and B: Western blot analysis against cell extracts containing wild type GST (lanes G) or antigen fused GST (lane N, P and V). (N, N1 of H1N1 influenza virus; P, PreS2 of HPV; V, VP1 of FMDV). For western blot analysis, the cell extracts were labeled with S5, SP1, or SV7 scFv, then detected with anti-His HRP antibody. Closed arrowhead in lanes N, P, and V indicate protein bands of viral antigenic peptide fused GST. Open arrowheads in lanes G indicate protein bands of wild type GST. Arrows in lanes N and P indicate the possible degraded forms of antigen-fused GST. (TIF) [file pone.0108225.s008.tif]
