# Supplementary material for: Rapid Isolation of Antibody from a Synthetic Human Antibody Library by Repeated Fluorescence-Activated Cell Sorting (FACS)
Source: PLoS One. 2014 Oct 10;9(10):e108225. doi: 10.1371/journal.pone.0108225 (PMC4193741; doi:10.1371/journal.pone.0108225)
Supplement: Table S1 — Bacterial strains and plasmids used in this study. (DOCX) [file pone.0108225.s009.docx]

**Table S1.** **Bacterial strains and plasmids used in this study**

| Strain or Plasmid | Relevant Characteristics | Reference or Source |
| --- | --- | --- |
| *E. coli* strains | | |
| Jude-1 | [(DH10B F′::Tn10 (Tetr)] | [32] |
| HM130 | KS272 *degP ptr ompT tsp eda* | [21] |
| Plasmids | | |
| pMoPac1 | 6.0 kb; Cm^r^, lac promoter | [33] |
| pUC19 | 2.7 kb; Amp^r^, lacZα | New England Biolabs^a^ |
| pMoPac16 | 7.4 kb; Amp^r^, lac promoter, pelB signal sequence, skp | [33] |
| pMoPac16-MBP | 8.6 kb; Amp^r^, lac promoter, malE signal sequence, malE gene (MBP) with FlAsH recognition sequence | This study |
| pMoPac1-blac | 6.7 kb; pMoPac1 derivative containing β-lactamase gene (792 bp) from pUC19 | This study |
| pMoPac1-V_H_-blac | 5.1 kb; pMoPac1 derivative containing variable heavy chain library with β-lactamase gene (792 bp) from pUC19 | This study |
| pMoPac1-V_L_-blac | 5.0 kb; pMoPac1 derivative containing variable light chain library with β-lactamase gene (792 bp) from pUC19 | This study |
| pMoPac1- V_H_ - V_L_ -blac | 5.4 kb; pMoPac1 derivative containing scFv library with β-lactamase gene (792 bp) from pUC19 | This study |
| pMoPac16- V_H_ - V_L_ | 6.0 kb; pMoPac16 derivative containing scFv library | This study |
| pGEX-4T-1 | 4.9 kb; Amp^r^ lacI^q^, GST gene | GE Healthcare^c^ |
| pMoPac1-GST-N1 | 4.5 kb; pMoPac1 derivative containing GST-fused N1 antigen | This study |
| pMoPac1-GST-PreS2 | 4.5 kb; pMoPac1 derivative containing GST-fused PreS2 antigen | This study |
| pMoPac1-GST-VP1 | 4.5 kb; pMoPac1 derivative containing GST-fused VP1 antigen | This study |

^a^New England Biolabs, Beverly MA

^b^GE Healthcare Biosciences AB, Uppsala, Sweden
