# Supplementary material for: Rapid Isolation of Antibody from a Synthetic Human Antibody Library by Repeated Fluorescence-Activated Cell Sorting (FACS)
Source: PLoS One. 2014 Oct 10;9(10):e108225. doi: 10.1371/journal.pone.0108225 (PMC4193741; doi:10.1371/journal.pone.0108225)
Supplement: Table S2 — Primers used for construction of GST-fused antigens, sFGFP, MBP. (DOCX) [file pone.0108225.s010.docx]

**Table S2. Primers used for construction of GST-fused antigens, sFGFP, MBP**

| **Primer name** | **Sequence (5’ → 3’)** |
| --- | --- |
| GST-F | ATATATCATATGTCCCCTATACTAGGTTATTGGAA |
| GSTN1-R | ATAAGCTTTCACTACCACGGACGGTTAGAACCGTGCCAGTTGTCACGGCAATCCGATTTTGGAGGATGGT |
| GSTPreS2-R | ATAAGCTTTCACTAGCCGCCCGCCGGAAAATACAGGCCGCGCACGCGCGGATCCAGCAGCGCCTGATGAAAGGTGGTGCTGTTATCCGATTTTGGAGGATGGT |
| GSTVP1-R | ATAAGCTTTCACTATTTTTGTGCCAACACTTGCAAATCGCCACGTACATTTGTGACTGGATCCGATTTTGGAGGATGGTCG |
| MalE-F | GCATTCTAGATTGAACTTTAAGAAGGAGATATACATATGAAAATAAAAACAGGTGCACGC |
| MalE-R | GCATAAGCTTATCACGGTTCCATACAGCAGCCCGGACAGCAATTCAGAAAGTGATGATGGTGATGATGCTTGGTGATACGAGTCTGC |
