# Supplementary material for: Rapid Isolation of Antibody from a Synthetic Human Antibody Library by Repeated Fluorescence-Activated Cell Sorting (FACS)
Source: PLoS One. 2014 Oct 10;9(10):e108225. doi: 10.1371/journal.pone.0108225 (PMC4193741; doi:10.1371/journal.pone.0108225)
Supplement: Table S3 — Primers used for construction of synthetic antibody library. (DOCX) [file pone.0108225.s011.docx]

**Table S3. Primers used for construction of synthetic antibody library**

| **Primer name** | **Number** | **Sequence (5’ → 3’)** | |
| --- | --- | --- | --- |
| **Primers for the framework regions** | | | |
| H-1F | 1 | GATTGTTATTACTCGCGGCCCAGCCGGCCATGGCGGAAGTGCAGCTGCT | |
| H-1R | 2 | CCACCTGGCTGCACCAGGCCGCCGCCGCTTTCCAGCAGCTGCACTTC | |
| H-2F | 3 | GCAGCCAGGTGGTAGTCTGCGTCTGAGCTGCGCGGCGAGCGGCTTTACCTTTAG | |
| H-3F | 4 | TGGGTTCGTCAAGCACCAGGTAAGGGTCTGGAATGGGTG | |
| H-4F | 5 | TGCGGATAGCGTGAAGGGTCGCTTTACCATTAGCCGCGATAACAG | |
| H-4R | 6 | GGCTGTTCATTTGCAGATACAGGGTGTTTTTGCTGTTATCGCGGCT | |
| H-5F | 7 | CTGCAAATGAACAGCCTGCGCGCGGAAGATACTGCCGTGTACTACTGTGCACGT | |
| H-6F | 8 | TTTGATTATTGGGGGTTACCGTGAGTAG | |
| H-6R-GS | 9 | CTGCCCCCTCCACCACTCCCGCCACCTCCACTACTCACGGTAACCA | |
| H-7F | 10 | TGGAGGGGGCAGCGGCGGTGGaGGgAGtGGCCTCGGGGGCCGAATTCGCGGCC | |
| L-1F | 11 | GATTGTTATTACTCGCGGCCCAGCCGGCCATGGCGaGCGGCCGCGAGATTGTGCTGACCCAGAGC | |
| L-1R | 12 | CCGGAGACAGACTCAGGGTGCCCGGGCTCTGGGTCAGC | |
| L-2F | 13 | AGTCTGTCTCCGGGTGAACGTGCGACCCTGAGCTGTCGTGCAAGTCAA | |
| L-3F | 14 | CAGCAGAAGCCAGGCCAGGCGCCGCGCCTGCTGATCTAT | |
| L-4F | 15 | GGTATTCCAGATCGCTTTAGCGGTAGCGGTAGCGGC | |
| L-4R | 16 | TCCAGGCGACTAATGGTCAGCGTGAAATCCGTGCCGCTACCGC | |
| L-5F | 17 | CATTAGTCGCCTGGAACCGGAAGATTTTGCGGTGTATTATTGC | |
| L-5R | 18 | TGCGTTTAATTTCCACTTTGGTGCCCTGGCCAAA | |
| L-6R | 19 | GGCCGCGAATTCGGCCCCCGAGGCCGTCGACGATGCGTTTAATTTCCACTTTG | |
| **Degenerate primers for the complementarity-determining regions** | | | |
| H-2R-1 | 20 | | TTTGCGGTGTATTATTGCSMGVVSNNKNNKAGCNNKVBGNNKRYCTTTGGCCAGGGCA |
| H-2R-2 | 21 | | GCTTGACGAACCCAGYSMATMBMATAGBYGCTAAAGGTAAAGCCGCT |
| H-3R | 22 | | CACGCTATCCGCATAWTHGGTGYHGCYGYYGYYMNNGYHAATMNNGCTCACCCATTCCAGACC |
| H-5R-7 | 23 | | CTGGCCCCAATAATCAAAMNNMNNMNNMNNMNNMNNMNNACGTGCACAGTAGTA |
| H-5R-8 | 24 | | CTGGCCCCAATAATCAAAMNNMNNMNNMNNMNNMNNMNNMNNACGTGCACAGTAGTA |
| H-5R-9 | 25 | | CTGGCCCCAATAATCAAAMNNMNNMNNMNNMNNMNNMNNMNNMNNACGTGCACAGTAGTA |
| H-5R-10 | 26 | | CTGGCCCCAATAATCAAAMNNMNNMNNMNNMNNMNNMNNMNNMNNMNNACGTGCACAGTAGTA |
| H-5R-11 | 27 | | CTGGCCCCAATAATCAAAMNNMNNMNNMNNMNNMNNMNNMNNMNNMNNMNNACGTGCACAGTAGTA |
| H-5R-12 | 28 | | CTGGCCCCAATAATCAAAMNNMNNMNNMNNMNNMNNMNNMNNMNNMNNMNNMNNACGTGCACAGTAGTA |
| H-5R-13 | 29 | | CTGGCCCCAATAATCAAAMNNMNNMNNMNNMNNMNNMNNMNNMNNMNNMNNMNNMNNACGTGCACAGTAGTA |
| H-5R-14 | 30 | | CTGGCCCCAATAATCAAAMNNMNNMNNMNNMNNMNNMNNMNNMNNMNNMNNMNNMNNMNNACGTGCACAGTAGTA |
| H-5R-15 | 31 | | CTGGCCCCAATAATCAAAMNNMNNMNNMNNMNNMNNMNNMNNMNNMNNMNNMNNMNNMNNMNNACGTGCACAGTAGTA |
| H-5R-16 | 32 | | CTGGCCCCAATAATCAAAMNNMNNMNNMNNMNNMNNMNNMNNMNNMNNMNNMNNMNNMNNMNNMNNACGTGCACAGTAGTA |
| H-5R-17 | 33 | | CTGGCCCCAATAATCAAAMNNMNNMNNMNNMNNMNNMNNMNNMNNMNNMNNMNNMNNMNNMNNMNNMNNACGTGCACAGTAGTA |
| H-5R-18 | 34 | | CTGGCCCCAATAATCAAAMNNMNNMNNMNNMNNMNNMNNMNNMNNMNNMNNMNNMNNMNNMNNMNNMNNMNNACGTGCACAGTAGTA |
| H-5R-19 | 35 | | CTGGCCCCAATAATCAAAMNNMNNMNNMNNMNNMNNMNNMNNMNNMNNMNNMNNMNNMNNMNNMNNMNNMNNMNNACGTGCACAGTAGTA |
| L-2R-1 | 36 | | CCTGGCTTCTGCTGATACCAGTDCASATAGYYGSTAMYGHYTTGACTTGCACGACA |
| L-2R-2 | 37 | | CCTGGCTTCTGCTGATACCAGSYCASATAGYYGSTAMYGHYTTGACTTGCACGACA |
| L-3R | 38 | | AGCGATCTGGAATACCGSTCKSCMGKBTGCTGDYGBMATAGATCAGCAGGCG |
| L-6F-VVS | 39 | | TTTGCGGTGTATTATTGCSMGVVSNNKNNKAGCNNKVBGNNKRYCTTTGGCCAGGGCA |
| **Primers for the amplification of the variable heavy and light chain** | | | |
| Assembly-F | 40 | | GATTGTTATTACTCGCGG |
| Assembly-R | 41 | | GGCCGCGAATTCG |
| **Primers for the circular polymerase extension cloning (CPEC)** | | | |
| CPEC-F |  | | GATTGTTATTACTCGCGGCCCAGC |
| CPEC-R |  | | GGCCGCGAATTCGGCCC |
| **Primers for the β-lactamase gene** | | | |
| Betalactam-F |  | | AGCTAAGCTTTAGTGATTACCAATGCTTAATCAGTGAGG |
| Betalactam-R |  | | AGCTGCGGCCGCACACCCAGAAACGCTGGTG |
